# Sex and Tamoxifen confound murine experimental studies in cardiovascular tissue engineering

**DOI:** 10.1038/s41598-021-87006-3

**Published:** 2021-04-13

**Authors:** Kevin M. Blum, Lauren C. Roby, Jacob C. Zbinden, Yu-Chun Chang, Gabriel J. M. Mirhaidari, James W. Reinhardt, Tai Yi, Jenny C. Barker, Christopher K. Breuer

**Affiliations:** 1 Center for Regenerative Medicine, The Abigail Wexner Research Institute, Nationwide Children’s Hospital, Columbus, USA; 2 Department of Biomedical Engineering, The Ohio State University, Columbus, USA; 3 College of Medicine, The Ohio State University, Columbus, USA; 4 Biomedical Sciences Graduate Program, College of Medicine, The Ohio State University, Columbus, USA; 5 Department of Plastic and Reconstructive Surgery, The Ohio State University, Columbus, USA

**Keywords:** Biomedical engineering, Cardiovascular biology

## Abstract

Tissue engineered vascular grafts hold promise for the creation of functional blood vessels from biodegradable scaffolds. Because the precise mechanisms regulating this process are still under investigation, inducible genetic mouse models are an important and widely used research tool. However, here we describe the importance of challenging the baseline assumption that tamoxifen is inert when used as a small molecule inducer in the context of cardiovascular tissue engineering. Employing a standard inferior vena cava vascular interposition graft model in C57BL/6 mice, we discovered differences in the immunologic response between control and tamoxifen-treated animals, including occlusion rate, macrophage infiltration and phenotype, the extent of foreign body giant cell development, and collagen deposition. Further, differences were noted between untreated males and females. Our findings demonstrate that the host-response to materials commonly used in cardiovascular tissue engineering is sex-specific and critically impacted by exposure to tamoxifen, necessitating careful model selection and interpretation of results.

## Introduction

Tissue engineered vascular grafts (TEVGs) represent a promising development in vascular surgery^1^. These polymer-based conduits degrade over time and are replaced by native tissue, resulting in the formation of a functional blood vessel made from the patient’s own cells. However, the precise cellular mechanisms underlying the development of TEVG neotissue remain to be elucidated^1^. Genetic mouse models are a powerful tool to study these processes because of their ability to manipulate cellular pathways both temporally, through the use of tamoxifen- and diphtheria-induced models, and spatially, through the use of cell-specific models^2–4^.

Tamoxifen, a selective estrogen receptor modulator, has wide-spread use as a small molecule effector for inducible knock-out models^5^. This system works by fusing a Cre-recombinase to an estrogen receptor (Cre^ERT2^) that resides in the cytosol until activated. Activated CRE^ERT2^ then translocates to the nucleus, where Cre induces recombination and subsequent knock-out of any gene modified with flanking Lox-P sites^6^.

Direct tamoxifen binding can weakly activate estrogen receptors, however its metabolites, formed through CYP2D6 enzymes within the liver, are much more biologically active^7^. Recently, several studies have identified that tamoxifen may have a confounding impact on mouse models. A single low dose of tamoxifen in wild type male mice was shown to have long term effects on testes and endocrine function^8^. Tamoxifen also dampened the inflammatory response and accelerated skin wound healing in ovarectomized females^9^. In the liver, tamoxifen attenuated hepatotoxicity, increased levels of antioxidants, and increased the presence of immune cells^10^. While utilizing tamoxifen-driven Cre^ERT2^ mice in a unilateral uretal obstruction model, tamoxifen was found to confound experimental results^11^. Specifically, tamoxifen attenuated fibrosis in female kidneys, but not male kidneys, even if tamoxifen injections were halted 14 days prior to the renal injury. In contrast, for neurologic models tamoxifen treatment via oral or IP administration did not affect behavior, cell proliferation, cell survival, or dendritic arborization in male or female mice^12^.

It is not surprising that tamoxifen may have off-target effects. Estrogen signaling is highly interconnected with many cellular signaling pathways, including proliferation, invasiveness, and apoptosis, which have effects far beyond the target tumor in cancer therapy^13^. Selective estrogen receptor modulators such as tamoxifen are also known to have variable tissue-specific effects, acting as an antagonist in breast tissue, but as a partial agonist in bone, uterine, and heart tissue^14^. In humans, tamoxifen is primarily used in estrogen receptor positive breast cancer treatment for which it has a potent antitumor effect, but also has off-target effects including a pro-tumorigenic effect on endometrial tissue, and a predisposition to thrombotic events such as deep vein thrombosis^15^. Tamoxifen also leads to decreases in blood cholesterol, triglyceride levels, leukocyte levels, and platelet levels, and an increase the incidence of radiotherapy-induced lung fibrosis ^15–17^.

Estrogen has an important role in wound healing and fibrosis, inhibiting macrophages and downregulating pro-inflammatory phenotypes^18^. As an estrogen modulator, it is therefore unsurprising that tamoxifen has shown effects in many fibrosis models in both humans and animals. Tamoxifen treatment in humans decreases the formation of hypertrophic scars in vivo, and in vitro decreases human fibroblast contraction of collagen matrices^19,20^. In pigs, tamoxifen has also been shown to limit fibrosis in a common bile duct anastomosis model^21^. Tamoxifen treatment in rats decreased fibrosis in pulmonary and nephrosclerosis models^22,23^. In a mouse model or tubulointerstitial fibrosis, tamoxifen decreased fibroblast proliferation, extracellular matrix deposition, and inflammation ^24^. Effects on fibrosis are presumably through mediation of TGF-β signaling, a cytokine known to play an important role in fibrosis, scar formation, and wound healing, as well as neotissue formation in tissue engineering^25–27^.

To date, no studies have examined the effect of sex on TEVGs nor the effect of tamoxifen in the context of tissue engineering. In addition, while tamoxifen is widely used in laboratory conditions for conditional knock-out models, it is often assumed to be inert in the systems being studied; a potentially hazardous assumption considering the number of studies examining its effects directly. In order to evaluate the potential effect of sex on neotissue formation as well as confounding effects of tamoxifen, we implanted TEVGs in both sexes of mice with and without tamoxifen treatment and evaluated the resulting neotissue.

## Results

### Mouse survival and surgical outcomes

**Figure 1:**
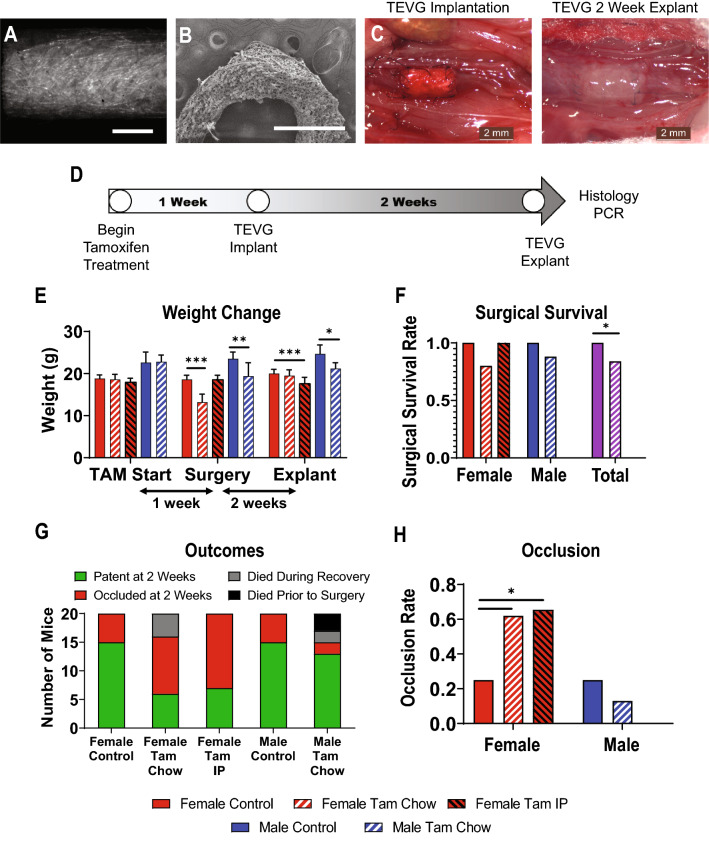
Experimental Overview and Outcomes. Transverse (**A**) and cross-sectional (**B**) view of the TEVG. (**C**) IVC interposition TEVG at implantation (Left) and 2-week explantation (Right). (**D**) Timeline of tamoxifen treatment, implant, and explant. (**E**) Survival and TEVG patency of all experimental animals. (**F**) Surgical survival rate of mice on control diet, tamoxifen chow, and IP tamoxifen injection. (**G**) Occlusion rate of TEVGs at explant. (**H**) Weight of animals measured at tamoxifen start date, date of surgery, and explant at two weeks post-implantation. Scale bars = 500 µm (**A** and **B**), 2 mm (**C**). N = 15–20 for weight measurement and compared statisticallyusing t-tests. N = 15–20 for occlusion rate and compared using Fisher’s exact test. *p < 0.05, **p < 0.01, ***p < 0.001.

In order to determine the sex-specific effects of tamoxifen on TEVGs, 8 to 10-week old male and female mice were administered a tamoxifen chow or control chow diet one week before TEVG implantation as an infrarenal inferior vena cava (IVC) interposition graft (Fig. 1A–D). Mice were kept on this diet throughout the evaluation period until sacrifice at two weeks post implantation. Two weeks was chosen as the study end date as we have previously reported this as a key time point for determining long-term TEVG outcomes in mice^28^.

Male mice, as expected, were larger than their age-matched female counterparts at each time point (Fig. 1E). On the date of surgery, one week after beginning treatment, tamoxifen chow led to a significant decrease in weight relative to control animals in both males and females (males 23.5 ± 1.6 vs 19.4 ± 3.2 g, *p* < 0.01; females 18.6 ± 1.0 vs 13.2 ± 1.9 g, *p* < 0.001), a known phenomenon due to food avoidance^29^. By the 2-week post-surgery explant date, the average body weight of female mice had recovered and was no longer different from untreated female mice (20.0 ± 1.0 vs 19.5 ± 1.4 g, *p*= 0.22). Interestingly, male mice on tamoxifen chow treatment remained at a lower weight than their controls at the time of sacrifice (24.7 ± 2.1 vs 21.2 ± 1.4 g, *p* < 0.001).

Tamoxifen treatment had an effect on surgical survival in both male and female mice (Fig. 1F). While untreated animals had 100% surgical survival, both male and female mice treated with tamoxifen experienced deaths occurring during the surgical recovery period (first 24 h post-implantation), although this difference was not significant for either sex individually (Fig. 1G). Interestingly, male mice on tamoxifen experienced deaths in the days prior to surgery, presumably related to their observed weight loss. Tamoxifen treatment had an effect on increasing the TEVG occlusion rate in female mice (from 25 to 62%, *p* < 0.05), however male mice were unaffected with equal occlusion rates in both groups (25% vs 13%, *p* = 0.67) (Fig. 1H).

Due to the significant weight loss seen in the tamoxifen-treated mice, an additional cohort of female mice was implanted with daily tamoxifen treatment delivered by intraperitoneal (IP) injection. Female mice administered with tamoxifen by IP injection did not experience weight loss and surgical survival matched untreated mice (100%), but the occlusion rate was not different from the tamoxifen chow group (65%, *p* < 0.05 vs female untreated controls). These observations suggest that weight loss was due to food aversion to the tamoxifen chow, surgical deaths may have been weight loss related, and occlusion was due to tamoxifen treatment and not due to weight loss, respectively.

### Neotissue formation

**Figure 2:**
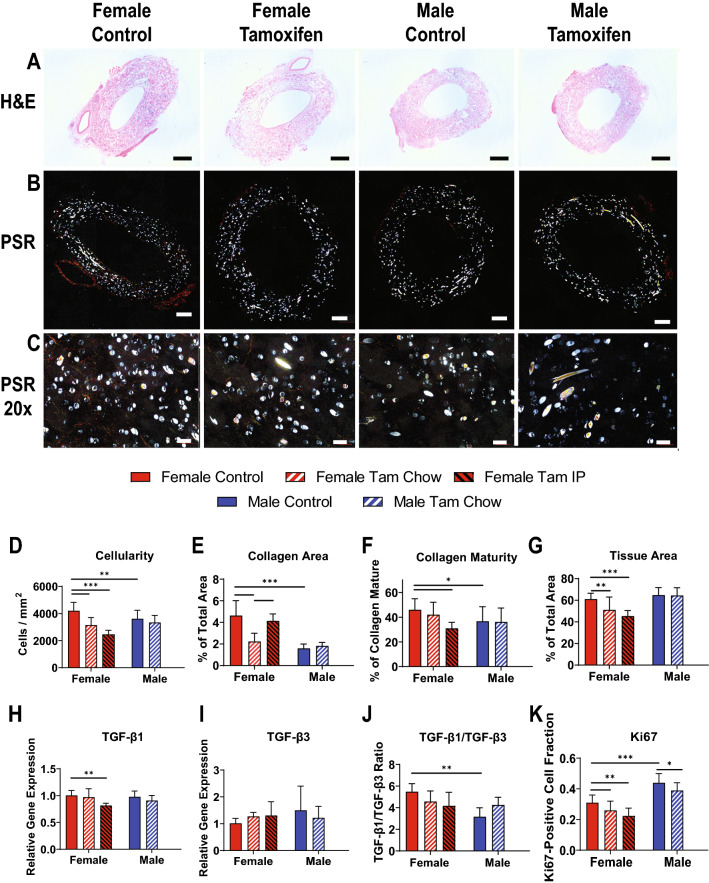
TEVG Neotissue Formation. (**A**) H&E imaging of TEVGs at the 2-week time point. (**B**) Picro-Sirius Red images under polarized light, with close-up of TEVG wall (**C**). White spots represent PGA fibers; red/orange coloration represents mature thicker collagen fibers and yellow/green coloration represents thinner, immature collagen fibers, respectively. IHC quantifications of cellularity (**D**), tissue area (**E**), collagen area (**F**), collagen maturity (**G**), and Ki67 (**H**). RT-qPCR quantifications of TGF-β1 (**I**), TGF-β3 (**J**), and ratio of TGF-β1 to TGF-β3 (**K**). Scale bars = 0.5 mm (**A**), 200 µm (**B**) 20 µm (**C**). N = 15–20 for histology measures (**D**–**G**), and N = 6 for RT-qPCR measures (**H**–**K**), all compared using t-tests. *p < 0.05, **p < 0.01, ***p < 0.001.

Explanted TEVGs were stained with hematoxylin & eosin (H&E) to evaluate cellularity and Picro-Sirius Red (PSR) for collagen (Fig. 2A–C). TEVGs implanted into untreated males had lower cellularity compared to untreated females (4206 ± 623 female vs 3611 ± 633 male cells/mm^2^, *p* < 0.01) (Fig. 2D), and markedly less collagen deposition (4.6 ± 1.4 vs 1.6 ± 0.4 area percent, *p* < 0.001) (Fig. 2E). Percentage of mature collagen was also decreased in untreated male mice compared to untreated females (46.0 ± 9.0 female vs 36.7 ± 11.8 male percent mature, *p* < 0.05) (Fig. 2F).

Tamoxifen had several effects on female mice. Cellularity was decreased in female mice on tamoxifen compared to female controls (4206 ± 623 control vs 3146 ± 568 chow vs 2436 ± 330 IP cells/mm^2^, *p* < 0.001 for IP and chow compared to control) (Fig. 2D). Tamoxifen decreased tissue area in females compared to untreated females (61.1 ± 5.4 control vs 51.1 ± 11.9 chow vs 45.3 ± 5.2 IP area percent, *p* < 0.01 control vs chow, *p* < 0.001 control vs IP) (Fig. 2G). Collagen area was decreased in females on tamoxifen chow compared to untreated females (4.6 ± 1.4 control vs 2.2 ± 0.8 chow area percent, *p* < 0.001) (Fig. 2E). Female mice on IP tamoxifen showed decreased collagen maturity (46.0 ± 9.0 control vs 31.0 ± 4.9 IP percent mature, *p* < 0.05) (Fig. 2F).

Due to known effects on wound healing and fibrosis, TGF-β1 and TGF-β3 gene expression were measured by reverse transcription quantitative real time polymerase chain reaction (RT-qPCR). TGF-β1 (Fig. 2H) and TGF-β3 (Fig. 2I) expression were not significantly different between males and females, although TGF-β1 expression was lower in IP tamoxifen treated females compared to untreated females (1.00 ± 0.09 control vs 0.82 ± 0.04 IP relative gene expression, *p* < 0.05). The ratio of TGF-β1 to TGF-β3, however, was lower in untreated males compared to untreated females (5.48 ± 0.75 female vs 3.16 ± 0.82 male TGF-β1 / TGF-β3 ratio, *p* < 0.01) (Fig. 2J). While there was a trend towards decreasing the TGF-β1/β3 ratio with tamoxifen treatment in females and increasing in males with tamoxifen treatment, the effects were not significant.

Cellular proliferation marker Ki67 (Fig. 2K) was higher in untreated males compared to untreated females (0.31 ± 0.05 female vs 0.44 ± 0.06 positive cell fraction, *p* < 0.001). In addition, tamoxifen treatment decreased Ki67 expression in both sexes (females 0.31 ± 0.05 control vs 0.26 ± 0.06 chow vs 0.22 ± 0.05 IP, *p* < 0.01; males 0.44 ± 0.06 control vs 0.39 ± 0.05 chow, *p* < 0.05).

### Estrogen receptor expression within Neotissue

**Figure 3:**
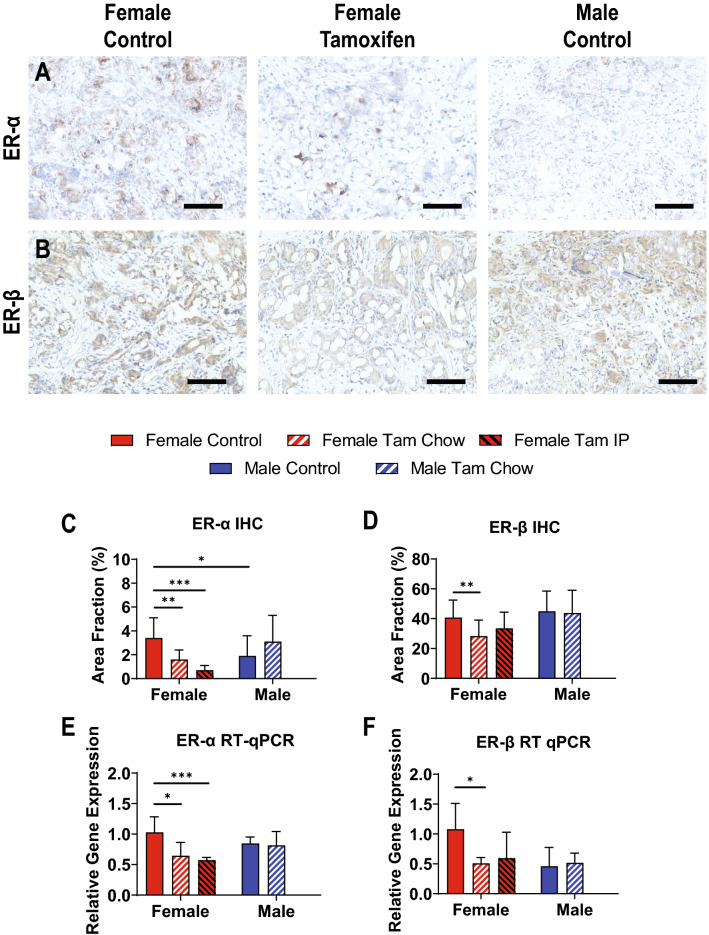
Estrogen Receptors Downregulated by Tamoxifen. Representative IHC of ER-α (**A**) and ER-β (**B**). IHC quantifications of ER-α (**C**) and ER-β (**D**). RT-qPCR analysis of ER-α (**E**) and ER-β (**F**). N = 15–20 for histology measures (**C**, **D**), and N = 6 for RT-qPCR measures (**E**, **F**), all compared using t-tests. Scale bars = 200 µm p < 0.05, **p < 0.01, ***p < 0.001.

Estrogen receptor alpha (ER-α) and estrogen receptor beta (ER-β) were downregulated by tamoxifen treatment in females as measured by both immunohistochemical staining (Fig. 3A–D) and RT-qPCR (Fig. 3E,F). ER-α histology staining was decreased in females with tamoxifen treatment and in untreated males compared to untreated females (female 3.4 ± 1.7control vs 1.6 ± 0.8 chow vs 0.7 ± 0.4 IP percent positive area, *p* < 0.01 control vs chow, *p* < 0.001 control vs IP; male 1.9 ± 1.7 vs 3.1 ± 2.2 control; *p* < 0.05 male vs female). ER-α gene expression was reduced in females with tamoxifen (female 1.03 ± 0.25 control vs 0.65 ± 0.22 chow vs 0.57 ± 0.05 IP relative gene expression, *p* < 0.05 control vs chow, *p* < 0.001 control vs IP). ER-β histology was only decreased in females (14 ± 5 control vs 8 ± 3 chow vs 7 ± 3 IP percent positive area, *p* < 0.001 for each vs control). ER-β gene expression was decreased in females with tamoxifen chow (1.08 ± 0.43 control vs 0.51 ± 0.11 chow relative gene expression, *p* < 0.05).

### Cellular make-up of Neotissue

**Figure 4:**
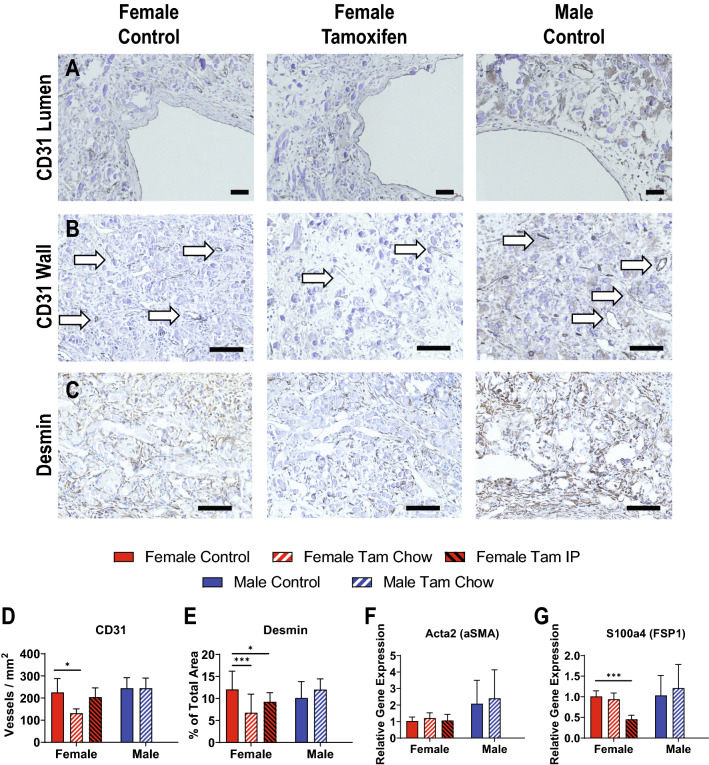
Tamoxifen Effects on Neotissue Cellular Make-Up. (**A**) All TEVGs were endothelialized by 2 weeks as demonstrated by CD-31 staining. (**B**) Walls of TEVGs contained vasa vasorum labeled with white arrows and quantified (**D**). (**C**) Fibroblast and smooth muscle marker desmin staining shown within the TEVG wall and quantified (**E**). RT-qPCR quantification of acta2 (**F**) and S100a4 (**G**) Scale bars = 50 µm (**A**), 200 µm (**B** and **C**). N = 15–20 for histology measures (**D**, **E**), and N = 6 for RT-qPCR measures (**F**, **G**), all compared using t-tests. *p < 0.05, **p < 0.01, ***p < 0.001.

At two weeks, all explanted TEVGs demonstrated complete endothelialization of the midgraft lumen (Fig. 4A). Within the walls of the TEVG, small vasa vasorum could also be seen by staining of endothelial cell junctions with anti-CD31 antibody (Fig. 4B). The density of these vessels was not different between males and females, but was decreased in females with tamoxifen chow treatment compared to untreated females (225 ± 63 vs 131 ± 20 vessels/mm^2^, *p* < 0.05) (Fig. 4D). Desmin, a marker of fibroblasts and immature smooth muscle cells, showed marked decrease in expression in females given IP or chow tamoxifen treatment (females 12.04 ± 4.16 control vs 6.76 ± 4.21 chow vs 9.22 ± 2.08 IP area percentage, *p* < 0.001 control vs chow, *p* < 0.05 control vs IP) (Fig. 4C,E). RT-qPCR for additional markers of fibrosis and smooth muscle cells, FSP-1 and αSMA, showed little to no differences between groups, with the exception of FSP-1 having decreased expression in tamoxifen IP females compared to untreated females (1.01 ± 0.13 control vs 0.45 ± 0.10 IP relative gene expression, *p* < 0.001) (Fig. 4F,G). Male gene expression of FSP-1 and αSMA also showed more within group variation than was observed for females.

### Inflammatory response to TEVG implantation

**Figure 5:**
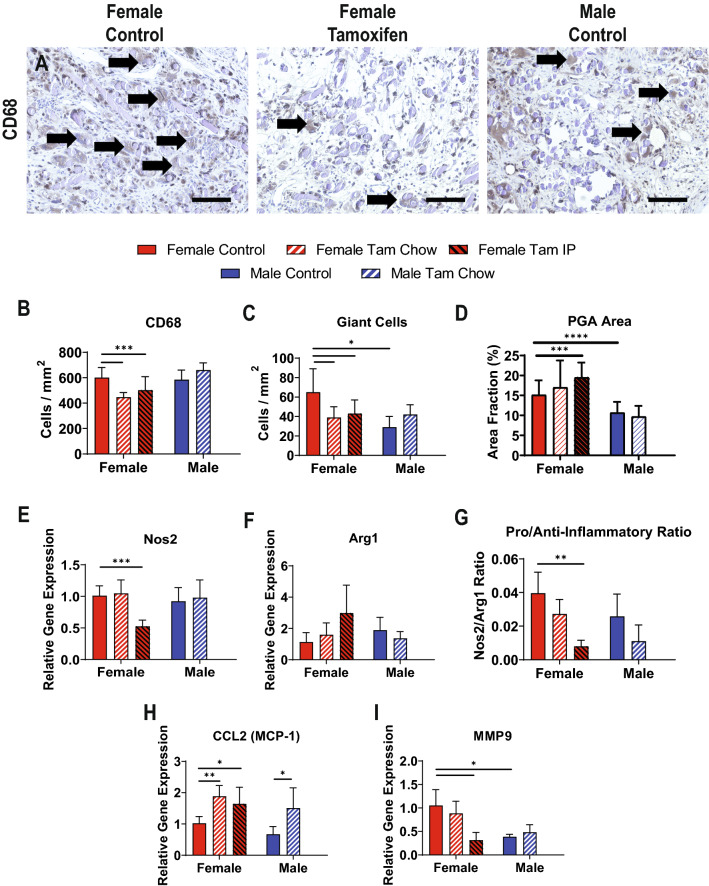
Tamoxifen Effects on Macrophage Response. (**A**) Representative imaging of CD68 staining, with giant cells labeled with black arrows. Total CD68 positive cells, including macrophages and giant cells quantified in (**B**) and giant cells alone quantified in (**C**). (**D**) Quantification of PGA polymer remaining in the wall of the TEVG 2 weeks after implantation. RT-qPCR quantification of pro-inflammatory NOS2 (**E**), anti-inflammatory Arg1 (**F**), and the ratio of the markers (**G**). RT-qPCR quantification of two markers of macrophage recruitment and function, CCL2 (**H**) and MMP9 (**I**). Scale bars = 200 µm. N = 15–20 for histology measures (**B**–**D**), and N = 6 for RT-qPCR measures (**E**–**I**), all compared using t-tests. *p < 0.05, **p < 0.01, ***p < 0.001.

CD68 was used as a pan-macrophage marker, staining both macrophages and foreign body giant cells (Fig. 5A). While the levels of CD68 expression were similar between untreated males and females, there was a marked difference in the formation of foreign body giant cells in response to the TEVG, with males having much lower giant cell formation (65 ± 24 vs 29 ± 11 giant cells/mm^2^, *p* < 0.05). Tamoxifen treatments in female mice decreased levels of both macrophages (601 ± 80 control vs 447 ± 36 chow vs 502 ± 106 cells/mm^2^, *p* < 0.001) and giant cells (65 ± 24 control vs 39 ± 11 chow vs 43 ± 14 IP cells/mm^2^, *p* < 0.05) (Fig. 5B,C).

The degradation of the TEVG biomaterial, as assessed by imaging for graft fibers under polarized light (Fig. 2B,C) was also different between sexes (Fig. 5D), with males having a lower percentage of residual polyglycolic acid fibers remaining at 2 weeks (female 15.15 ± 3.60 vs male 10.70 ± 2.68 area percentage, *p* < 0.0001). Females on IP tamoxifen, which demonstrated a decreased number of macrophages, also demonstrated a higher amount of residual graft material compared to female controls (15.15 ± 3.60 control vs 19.49 ± 3.72 IP area percentage, *p* < 0.001).

RT-qPCR for inflammatory M1 (NOS2) (Fig. 5E) and anti-inflammatory M2 (Arg1) (Fig. 5F) macrophage markers demonstrated no significant differences between groups other than between control female mice and female mice with IP tamoxifen. In addition, the NOS2/Arg1 ratio was less than 0.1 in all groups, suggesting a heavily anti-inflammatory macrophage phenotype (Fig. 5G). NOS2/Arg1 ratio in female mice on IP tamoxifen was significantly decreased (0.040 ± 0.013 control vs 0.008 ± 0.004 IP, *p* < 0.01) while control males trended towards a decrease relative to control females and females on tamoxifen chow trended towards a decrease compared to control females without statistical significance. Monocyte chemoattractant protein 1 (MCP-1) gene expression was increased in both male and female mice on tamoxifen relative to controls (females 1.02 ± 0.21 control vs 1.88 ± 0.34 chow vs 1.64 ± 0.53 IP relative gene expression, *p* < 0.01 control vs chow, *p* < 0.05 control vs IP; males 0.67 ± 0.25 control vs 1.51 ± 0.65 chow relative gene expression, *p* < 0.05 control vs IP) (Fig. 5H), notable as macrophage levels were not increased in any treatment groups. MMP-9 gene expression was decreased in males relative to females (1.05 ± 0.33 females vs 0.39 ± 0.05 males relative gene expression, *p* < 0.05), and decreased in females on IP tamoxifen relative to control (1.05 ± 0.33 control vs 0.31 ± 0.17 IP relative gene expression, *p* < 0.05) (Fig. 5I).

## Discussion

Improving our understanding of the biomechanical, cellular, and molecular pathways underlying neotissue formation in TEVGs holds the key to improving their performance through rational design. A lack of effective and correlative biological implantation models hinders the translation of tissue engineering findings, as the differences in geometry, mechanics, and biological signaling between humans and research animals must be considered^30^. A history of empiric design changes and studies using a small number of animal models has made comparisons between studies difficult and further hinders a mechanistic understanding of tissue engineering paradigms^31^. Fine-tuning of mechanical and microstructural scaffold parameters can vastly alter biological outcomes^32,33^. However, even when engineering factors affecting scaffold creation are tightly controlled, biologic factors such as sex and age can play a significant role in altering outcomes in TEVGs, demonstrating a need for careful and detailed evaluation of biological experimental models and their baseline assumptions and limitations^34,35^. In this study, we have shown multiple effects of sex on a cardiovascular tissue engineering model, as well as sex-specific effects of tamoxifen. To our knowledge this is the first study to directly compare the outcomes of in situ TEVGs in male and female animals. Sex-specific differences in untreated mice, particularly in cellularity, deposition of neotissue, development of giant cells in reaction to biomaterial implantation, and differences in rate of biomaterial degradation have strong consequences towards the development of ideal tissue engineering solutions.

While the precise mechanisms at play in the sex differences in tissue engineering outcomes and in the broader field of wound healing are complex, previous studies have shown inherent differences in inflammation and wound healing properties between sexes and have also demonstrated relation to levels of estrogens and androgens^36,37^. Human studies have shown differences in estrogen signaling as well as in inflammatory state between the sexes in untreated animals. Female patients are more susceptible to inflammatory and autoimmune disorders such as asthma, and higher numbers of macrophages and giant cells, as well as a stronger inflammatory response are typically seen in women^38^. Studies in mice have demonstrated that there are differences between males and females in peritoneal macrophage behavior and rate of replenishment, which is higher in males^39^. This is particularly of importance to the current study as the TEVG is implanted in the peritoneal cavity. Additionally, in human macrophages, ER-α and ER-β are present in males and females, and are higher in males^40^. Levels of monocytes and macrophages in blood and tissues are shown to be different across males and females, and across various mouse strains used in research^41^. Prior studies have further demonstrated that estrogen exerts different effects on males versus females. Studies in mice found that estrogen given to ovarectomized mice can reduce MCP-1 and leukocyte infiltration, suggesting that estrogen acts as a reprogramming switch for macrophages to turn from M1 to M2 by acting through ER-α^42^. The same study found that post-menopausal women have increased M1 response, with similar M2 response, shifting the M1/M2 balance^42^. In the present study the M1/M2 ratio was found to heavily favor the M2 anti-inflammatory macrophage phenotype, notable as the ratio of pro- to anti-inflammatory macrophages has been shown to be critical to the wound healing and tissue regenerative pathways^43,44^.

Results in this study related to the inflammatory response further this understanding and go on to suggest that macrophages and monocytes of males and females respond differently to implanted biomaterials, an important consideration for evaluation of biomaterials as well as their translation to the clinic. Females create more foreign body giant cells and produce more collagen. Males, on the other hand, degrade the implanted biomaterial much more rapidly despite similar levels of macrophages to females. The large difference in the inflammatory response between sexes compared to similar M1/M2 ratios seen in this study may suggest that additional factors related to macrophage and monocyte function in response to biomaterials should be investigated in future studies. The differences seen in the collagen deposition and resorption of scaffold polymer seen between the groups suggests that mechanical differences may be arising between neovessels. Further investigation into the mechanical properties of developing neotissue may shed further light on the processes underlying its formation. Previous work evaluating the abdominal aortas in humans found age and sex-dependent differences in native abdominal aortas, with males having stiffer vasculature with greater age-related loss of stiffness^45^.

In addition to sex differences in tissue regeneration and remodeling, we showed that tamoxifen treatment alters the host response to TEVGs. Tamoxifen has previously been shown to prevent endothelial cell migration and proliferation in a rat model, and while estrogen can accelerate the re-endothelialization of denudated carotid arteries, tamoxifen does not have this effect^46,47^. This is corroborated by our findings in this work in female mice, where Ki67-labeled proliferation and CD31-labeled endothelial cell expression within TEVGs were decreased in response to Tamoxifen treatment. Interestingly, male mice treated with tamoxifen also had a decrease in Ki67 expression.

In evaluation of neotissue formation and remodeling in tissue engineering systems, it is important to note that these processes are dynamic, and show a large degree of variation over time^48–50^. As this evaluation focused specifically on the two-week time point, which has previously been shown to be a key time point in evaluation of our TEVGs in previous murine models, it is possible that the differences seen may be due to differences in kinetics of cellular and molecular processes, which may or may not converge over time^28,51–53^. As such, further experimentation evaluating the time courses of these differences would shed greater light on the mechanisms at play.

Throughout early experiments, the significant weight loss associated with the tamoxifen chow treatment raised concern of weight loss and malnutrition being causal of the findings in this study, as opposed to effects of tamoxifen directly, as decreased cellularity and increased inflammation have been seen in these states^54,55^. Malnutrition has been shown to interact with many important wound healing pathways, including upregulation of IL-6 and TNF-a, as well as inhibiting collagen synthesis^56^. As an alternative to tamoxifen chow, mouse studies have previously demonstrated that IP tamoxifen has little effect on weight^57^. Based on these observations, we revised our experimental design by including treatment with tamoxifen by IP injection. Tamoxifen IP injection demonstrated similar outcomes to tamoxifen chow, but without weight loss, allowing us to conclude that differences were due to tamoxifen.

While results between tamoxifen chow or daily tamoxifen IP injection are similar in females, there were several differences noted, mainly in effect size. These differences may be due to differences in bioavailability of tamoxifen for each dosing strategy, or by the differences in dosing between a controlled IP injection and an ad libitum diet of tamoxifen chow. In addition, there is the possibility that the weight loss seen in the tamoxifen chow group exhibited additional effects on top of the tamoxifen itself, potentiating some effects and antagonizing others.

It is relevant to note that, despite widespread clinical use, the mechanism of action of tamoxifen on biological systems is debated. While some studies have demonstrated direct effects through canonical estrogen receptor signaling pathways, separate studies have shown biological effects with no interaction through the canonical pathways^58–60^. It is likely that tamoxifen, as a steroid modulator, has multiple effects that can be enacted through a number of biological pathways within and outside of traditional canonical signaling. Additionally, tamoxifen has been shown to alter TGF-β signaling in some models, including differences in the TGF-β1 / TGF-β3 ratio. This is particularly relevant to tissue engineering, as fetal scarless healing is noted to have low TGF-β1 and high TGF-β3^61^. Topical tamoxifen has seen some clinical success in reducing keloid scar formation, with a suggested mechanism of a reduction in TGF-β1 levels^62^.

Despite tamoxifen’s long-standing use in the laboratory as a Cre-Lox inducer and the prevailing thought that tamoxifen is essentially inert in the mouse model, multiple recent studies, including this one, have found confounding effects of tamoxifen on biological systems. In one study, tamoxifen IP injection in male mice attenuated CCL4-induced hepatotoxicity, downregulated CYP2E1 activity in the liver, and increased levels of antioxidants including catalase and superoxide dismutase. Tamoxifen also increased the presence of resident macrophages and recruitment of immune cells to necrotic areas of the liver^10^. IP tamoxifen increased browning of adipose tissue in female mice, but not male mice^57^. Careful consideration of these effects, and perhaps more that have not yet been elucidated, should be given when choosing to use tamoxifen in experimental models.

Overall, this study demonstrated the complicated biological mechanisms at play in tissue engineering constructs. Even in syngeneic animals, the effects of sex cannot be ignored, and in fact can provide insight into important biological differences and potential avenues to improve tissue engineering outcomes. These innate differences due to sex as well as differences which arise due to changes in hormone status, which can be altered with age as well as disease state or medication use, may prove to be an important consideration for predicting patient outcomes and evaluating potential applications of tissue engineering technologies. The use of any treatment on an animal or patient, in this case tamoxifen, can also have many unanticipated off-target effects on a tissue engineering system. The dosing route of the drug may also be an important factor in determining any confounding effects. Careful development of experimental design and thoughtful analysis of resulting outcomes will be crucial for the continued development of the field of tissue engineering, and determination of the biological mechanisms at play.

## Methods

All surgeries, procedures, and experiments involving the use of animals in this study were done an accordance with relevant guidelines and regulations with approval from the Abigail Wexner Research Institute (AWRI) at Nationwide Children's Hospital Institutional Animal Care and Use Committee (IACUC) (Protocol AR12-00,075). All animal experiments were performed in accordance with relevant guidelines and regulations and our animal study reporting adheres to the ARRIVE guidelines.

### Scaffold creation

Murine TEVGs were prepared as described previously^63^. Briefly, nonwoven polyglycolic acid (PGA) felt with a fiber diameter of 16 µm was wound around a 19G needle to set the internal TEVG diameter, and inserted into a plastic mold to set the outer diameter. A 50:50 molar mixture of poly-L-lactide and poly-caprolactone (PCLA), 5% by mass/volume in dioxane was then used as a sealant for the TEVGs. Scaffolds were then frozen to -80C and lyophilized overnight to remove residual solvent.

### Animal treatments, TEVG implantation, and harvest

Male and female C57BL/6 mice aged 8–10 weeks were implanted with TEVGs as IVC interposition grafts using standard microsurgical techniques^51^. Briefly, after appropriate anesthesia with an intraperitoneal injection consisting of ketamine (100 mg/kg) and xylazine (10 mg/kg), a midline laparotomy incision was made, and a self-retaining retractor was inserted. The intestines were wrapped with gauze that was moistened in sterile saline. The aorta and IVC were separated, 2 microclamps were placed on the IVC, and the IVC was then transected between the microclamps. The graft was implanted as an IVC interposition graft with proximal and distal end-to-end anastomosis using a sterile 10–0 suture. Following skin closure, animals were moved to a recovery cage with a warming pad until they regained full mobility. Upon recovery, the mouse was returned to a new cage, and pain medication (ibuprofen) was provided in the drinking water for 48 h^53^.

Tamoxifen was administered in chow (Envigo, Huntingdon, UK), beginning one week before implantation^5^. Mice were kept on the chow throughout the experimental study until endpoint. Mice were weighed on the day tamoxifen treatment began, the day of surgery, and the day of explant (POD 14).

To evaluate the administration route-specific effects of tamoxifen, an additional cohort of female mice was treated with 75 mg/kg body weight of tamoxifen dissolved in peanut oil by IP injection. Injections were done daily following the same timeline as the chow group in order to approximate the same dosing regimen.

A total of twenty mice were used per experiment group (male control, male tamoxifen chow, female control, female tamoxifen chow, female tamoxifen IP).

Two weeks after graft implantation (three weeks total after beginning tamoxifen treatment), mice were euthanized by deeply anesthetizing via intraperitoneal injection of an overdose of ketamine (200 mg/kg) and xylazine (20 mg/kg). Subsequently, the chest was cut open, and an incision was made on the right atrium; the mouse was systemically perfused from the left ventricle with 20 ml of 0.9% saline. Grafts were explanted surgically and divided in half, with one half being formalin fixed for histology, and the other half being snap-frozen in liquid nitrogen for RT-qPCR.

### Histology

Explanted TEVG sections for histology were fixed overnight in formaldehyde at 4 °C and transitioned to 70% ethanol. Samples were then paraffin embedded and sectioned at 4 µm. Samples were stained with H&E or Picro-Sirius Red, or left bare for immunohistochemistry. Immunohistochemistry sections were deparaffinized, rehydrated and blocked for endogenous peroxidase activity (3% H2O2 in H2O) and nonspecific background staining (3% normal goat serum in Background Sniper, BioCare Medical, CA, USA). Antigen retrieval was performed with citrate buffer (pH 6.0) or Tris–EDTA (pH 9.0) in a pressure cooker for 10 min and slides were incubated for 30 min at room temperature with primary antibodies. Primary antibodies included desmin (ab1520, abcam), CD31 (ab28364, abcam), CD68 (ab125212, abcam), Ki67 (ab15580, abcam), ER-α (ab271827, abcam), and ER-β (ab3576, abcam). Primary antibody binding was detected by subsequent incubation with species appropriate biotinylated IgG (Vector, CA, USA), followed by streptavidin-horse radish peroxidase (Vector) and chromogenic development with 3,3-diaminobenzidine (Vector). Tissue sections were counterstained with Gill’s hematoxylin (Vector), and slides were dehydrated and cover slipped. Slides were imaged on a Zeiss Axio Observer Z1 inverted microscope (Zeiss, Germany) and quantified using ImageJ (NIH, USA). Images were de-identified and randomized prior to quantification to prevent bias. Collagen maturity was determined as percent of collagen fibers which demonstrated red/orange birefringence under polarized light. Giant cells were counted from CD68 + cells containing multiple nuclei, indicative of fused macrophages that are the hallmark of giant cells^64^.

### Reverse transcription quantitative real time polymerase chain reaction

Explanted TEVG sections were snap frozen and stored at -80C prior to transitioning to RNA*later*-ICE (Invitrogen, AM7030) according to manufacture protocol. Samples were homogenized with a Qiagen Tissuelyser II and processed using an RNeasy Mini Kit (Qiagen, #74,104). On column DNA digestion was performed with an RNase-Free DNase Set (Qiagen, #79,254). Purified RNA was analyzed on a Nanodrop 2000c for concentration and 260/280 ratio greater than 1.8. Conversion of RNA to cDNA was performed with the High-Capacity RNA-to-cDNA kit (Applied Biosystems, #4,388,950). Custom-designed Taqman Array 96 well plates were used with the following hybridization (Taqman) probes: 18 s rRNA (Hs99999901_s1), Actb (Mm02619580_g1), Hprt (Mm03024075_m1), B2m (Mm00437762_m1), Tgfb1 (Mm01178820_m1), Tgfb3 (Mm00436960_m1), NOS2 (Mm00440502_m1), Arg1 (Mm00475988_m1), Acta2 (Mm00725412_s1), S100a4 (Mm00803372_g1), Esr1 (Mm00433149_m1), Esr2 (Mm00599821_m1), Ccl2 (Mm00441242_m1), Mmp9 (Mm00442991_m1), F3 (Mm00438855_m1), and Igf1 (Mm00439560_m1). A gene maximation plate design was used loading equal amounts of cDNA for each sample with Taqman Fast Advanced Master Mix (Applied Biosystems, #4,444,556)^65^. Plates were run on an Applied Biosystems 7500 Real-Time PCR System. A total of 4 potential reference genes, 18srRNA, Actb, Hprt, and B2m, were run for every sample to determine the most stably expressed gene for the relative quantification. Resulting CT values for these 4 reference genes were analyzed utilizing the online tool RefFinder, a combination of BestKeeper, NormFinder, Genorm, and the comparative Delta-Ct method^66^. B2m was determined to be the most stably expressed reference gene across all samples and was subsequently utilized for delta-delta CT relative quantification calculations.

### Statistical analysis

Data analysis was performed using GraphPad Prism 8.0 software (GraphPad, CA USA). Histology imaging for cellular markers was performed on 4 independent regions within each histological section, and averaged as technical replicates for comparison. RT-qPCR data was analyzed using delta-delta CT values, taken as the average of 2 technical replicates. Data was compared with t-tests between groups with experimental relevance (female control vs female tam chow, female control vs female tam IP, female control vs male control, male control vs male tam chow). Surgical survival and occlusion rate were compared using Fisher’s exact test.
